# Black phosphorus quantum dots camouflaged with platelet-osteosarcoma hybrid membrane and doxorubicin for combined therapy of osteosarcoma

**DOI:** 10.1186/s12951-023-02016-9

**Published:** 2023-07-28

**Authors:** Yanlong Xu, Longhai Du, Binxu Han, Yu Wang, Jichang Fei, Kuo Xia, Yonghua Zhai, Zuochong Yu

**Affiliations:** 1 Department of Orthopedics, Jinshan Hospital, Fudan University, Longhang Road 1508#, Jinshan District, Shanghai, 201508 People’s Republic of China; 2 Department of Orthopedics, Nanping First Hospital of Fujian Medical University, Fujian, People’s Republic of China; 3 Changhai Clinical Research Unit, Shanghai Changhai Hospital, Naval Medical University, Shanghai, People’s Republic of China

**Keywords:** Photothermal therapy (PTT), Black phosphorus quantum dots (BPQDs), Hybrid membrane, Osteosarcoma (OS), Doxorubicin (DOX)

## Abstract

**Background:**

Osteosarcoma (OS) is the most prevalent primary malignant bone tumor. However, single-agent chemotherapy exhibits limited efficacy against OS and often encounters tumor resistance. Therefore, we designed and constructed an integrated treatment strategy of photothermal therapy (PTT) combined with chemotherapy and used a surface-encapsulated platelet-osteosarcoma hybrid membrane (OPM) that enhances circulation time and enables OS-specific targeting.

**Results:**

The OPM functions as a shell structure, encapsulating multiple drug-loaded nanocores (BPQDs-DOX) and controlling the release rate of doxorubicin (DOX). Moreover, near-infrared light irradiation accelerates the release of DOX, thereby extending circulation time and enabling photostimulation-responsive release. The OPM encapsulation system improves the stability of BPQDs, enhances their photothermal conversion efficiency, and augments PTT efficacy. In vitro and ex vivo experiments demonstrate that BPQDs-DOX@OPM effectively delivers drugs to tumor sites with prolonged circulation time and specific targeting, resulting in superior anti-tumor activity compared to single-agent chemotherapy. Furthermore, these experiments confirm the favorable biosafety profile of BPQDs-DOX@OPM.

**Conclusions:**

Compared to single-agent chemotherapy, the combined therapy using BPQDs-DOX@OPM offers prolonged circulation time, targeted drug delivery, enhanced anti-tumor activity, and high biosafety, thereby introducing a novel approach for the clinical treatment of OS.

**Supplementary Information:**

The online version contains supplementary material available at 10.1186/s12951-023-02016-9.

## Introduction

Osteosarcoma (OS) is the third most prevalent malignancy in children and adolescents, following leukemia and lymphoma [1]. The current standard treatment for OS involves a combination of surgery and chemotherapy [2]. Although the application of these treatment protocols has significantly improved OS outcomes, the 5-year survival rate remains at most 70%. Moreover, for patients who experience recurrence or metastasis, the 3-year survival rate is a mere 20% [3]. The development of chemotherapy resistance plays a crucial role in the poor prognosis of OS patients [4]. Furthermore, the OS genome displays a high level of complexity and heterogeneity [5, 6], making it unlikely that single-agent chemotherapy will effectively target all cellular subtypes of OS. Multiple clinical studies demonstrate that combined oncologic therapy can effectively address the poor efficacy associated with single-agent chemotherapy. Such therapies can selectively eliminate tumor cells while minimizing damage to normal tissues and reducing chemotherapy resistance. Hence, there is an urgent need in the clinical field to develop novel, safe, efficient combined therapies.

Doxorubicin (DOX)-based chemotherapy regimens are the standard of care for OS [7, 8]. Separately, photothermal therapy (PTT), a novel tumor treatment modality, has recently attracted much attention due to its high selectivity and minimal side effects [9]. In PTT, a photosensitizer increases the local cell and tissue temperature. When the temperature rises to 42 °C, the tissue cells undergo protein aggregation and become more susceptible to chemotherapy and radiotherapy [10]. When temperatures rise to 48 °C, tissues may suffer irreversible damage and eventually undergo necrosis [11]. Furthermore, the reactive oxygen species (ROS) generated during PTT can inhibit drug efflux P-glycoprotein pumps in multidrug-resistant tumor cells and enhance the chemotherapeutic efficacy [12]. Chemotherapeutic drugs can, in turn, improve the limitation of light penetration in PTT and increase tumor cell sensitivity to thermotherapy or ROS [13]. Combining the two therapies can produce a synergy and an enhanced therapeutic effect [14]. However, conventional chemotherapeutic drugs have drawbacks such as high toxicity and susceptibility to rapid blood clearance [15]. Therefore, choosing a photosensitizer suitable for drug delivery can contribute toward better exploiting the combined therapeutic effect of chemotherapy and PTT.

Black phosphorus (BP), a phosphorus isomer, is an emerging nanomaterial in cancer therapy because of its excellent drug delivery capability, biocompatibility, in vivo biodegradability, and excellent photothermal/photodynamic properties [16–18]. Black phosphorus quantum dots (BPQDs) are ultrasmall derivatives of BP nanosheets that exhibit a superior extinction coefficient and high photothermal conversion efficiency compared to that of BP [19, 20]. The irradiation of BPQD dispersions using 808-nm near-infrared (NIR) light increased their temperature from 18.3 °C to 50.1 °C within 7 min [21] and caused irreversible necrosis of tumor cells. Further, BPQDs degrade more rapidly under acidic conditions, thus accelerating the release of anti-tumor drugs in an acidic tumor microenvironment [22]. Numerous studies have shown that nano-sized BPs have a large surface area due to their pleated structure, enabling them to have a high drug-loading capacity. In addition, as a drug carrier with a negative surface charge, BP can load numerous types of drugs with positive surface charge onto the surface of BP materials by electrostatic adsorption, and BPQDs, as a type of BP, have better drug loading performance because of their large surface area-to-volume ratio [23]. However, BPQDs become susceptible to oxidative degradation in the presence of oxygen and water for a long time. Polymer surface modification or BPQD coating reagents can significantly improve their stability, and biofilm encapsulation systems have shown great potential as a novel surface modification method.

Biofilm systems are widely used because they are not cleared by the immune system and exhibit noncytotoxicity and high bioavailability [24, 25]. Platelet surfaces have a long half-life because they contain CD47, which can interact with signaling regulatory proteins on immune cells to inhibit immune clearance [26]. One of the most unique properties of cancer cells as part of membrane-encapsulated nanoparticle technology is the ability to isotype targeting [27], allowing for targeted drug delivery to the tumor site while reducing systemic toxicity. In this study, we fused the osteosarcoma cell membrane (OCM) with a platelet cell membrane (PM) to construct a platelet-osteosarcoma hybrid membrane (OPM) that can achieve immune escape, long blood circulation time, and OS isotype targeting.

**Fig. 1:**
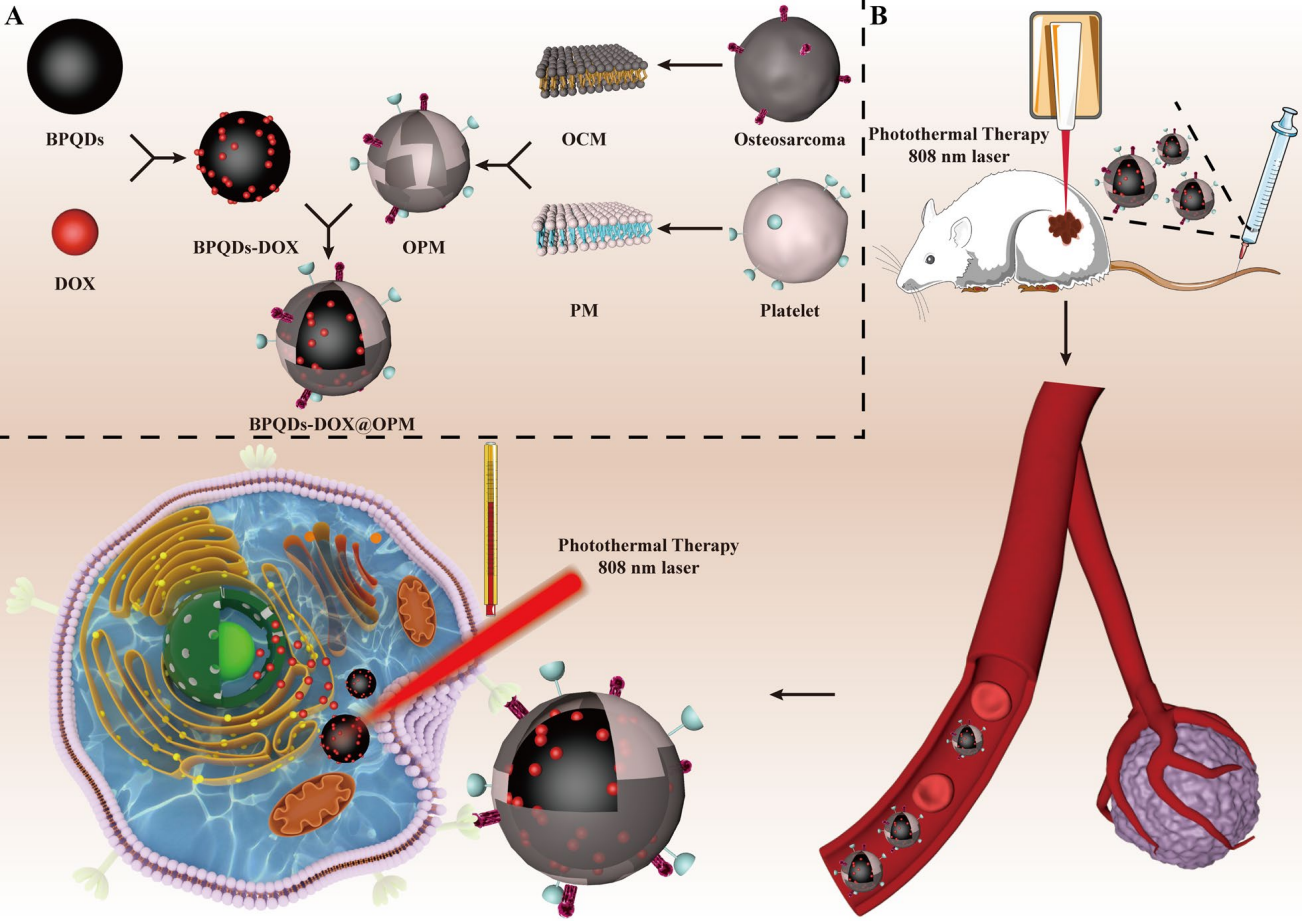
Schematic diagram of the role of OPM camouflaged with BPQDs-DOX in combined treatment of OS. **A** Preparation process of BPQDs-DOX@OPM drug delivery system. **B** BPQDs-DOX@OPM has a long circulation time and OS homotypic targeting ability to deliver drugs to the tumor site, thus enhancing the efficacy of chemotherapy combined with PTT

In this study, we used BPQDs, a photosensitizer with high drug loading performance, to load DOX. Then, we used OPM to encapsulate the surface of BPQDs-DOX to confer a long circulation time and OS isotype targeting. The biocompatibility, targeting, and combined therapeutic effects of the drug delivery system were verified through in vivo and in vitro experiments. This paper presents the preparation, characterization, and in vitro and in vivo performance validation of the BPQDs-DOX@OPM combined drug delivery system developed to realize combined chemotherapy and PTT for clinical OS treatment (see Fig. 1).

## Materials and methods

### Materials

A BPQD dispersion was purchased from XFNANO Ltd. (China). Doxorubicin hydrochloride and floral cyanine dye cy5 were purchased from Aladdin Ltd. (China). CD47 primary antibody and epidermal growth factor receptor (EGFR) primary antibody were purchased from CST. (USA). Fetal bovine serums were purchased from Gibco. (USA). Polycarbonate porous membrane filters were purchased from Whatman. (UK). A cell membrane far-infrared fluorescence staining kit (DiD) was purchased from Biyuntian Biotechnology. (China). An Annexin V-FITC/7-AAD apoptosis assay kit was purchased from Sino Biological. (China). M5 Matrigel matrix gel (MF232-01) was purchased from Beijing Polymeric Biotechnology Co. (China) (see Additional file 1).

### Cells and mice

Human osteosarcoma cell line (Saos-2), human brain microvascular endothelial cell line (HBMEC), and mouse embryonic fibroblast cell line (3T3) were purchased from the Shanghai Chinese Academy of Sciences cell bank, and mouse macrophage cell line (RAW264.7) was purchased from Nanjing KEBAI Biotechnology Co. Balb/c nude mice (females; weight: 20–22 g; age: 4–5 weeks) were provided by Beijing Viton Lever Laboratory Animal Technology Co.

### Extraction of cell membranes

Platelet cell membranes were extracted by a repeated freeze-thawing method [28]. Platelet-rich plasma was added to phosphate-buffered saline (PBS) containing 1 mmol/L ethylenediaminetetraacetic acid (EDTA) and 2 mmol/L prostaglandin E1, and PBS containing 1 mmol/L EDTA and protease inhibitor was added after centrifugation at 3000 rpm to prepare the platelet membrane suspension. This suspension was frozen at − 80 °C and thawed at 37 ℃, and this process was repeated three times. Subsequently, the cell membranes were extracted and dispersed in PBS-containing protease inhibitor, and platelet vesicle membranes were obtained after ultrasonication in a water bath at 100 W and 42 kHz for 6 min.

The OS cell membranes were extracted according to the ultrasonic fragmentation method. Digested Saos-2 cells were added to a buffer containing 75 mM sucrose, 225 mM mannitol, 30 mM Tris–HCl, 0.5 mM EDTA and 1% (v/v) protease inhibitor and mixed thoroughly, after which the cells were sonicated and fragmented using a probe sonicator (30 W, 3 min). After centrifuging the suspension (800 g, 15 min) the supernatant was taken, and the supernatant obtained was centrifuged again (10,000*g*, 30 min) and finally dispersed in PBS buffer containing protease inhibitor to make OCM suspension.

### Preparation of BPQDs-DOX

DOX was mixed with BPQDs for 24 h and then placed in a 2 kD dialysis membrane to remove the free DOX. The dialyzed sample was used to detect the DOX concentration and to calculate the encapsulation rate and drug loading capacity of DOX. The DOX encapsulation rate was calculated as EE% = (DOX input—DOX recovery)/DOX input × 100%. The drug loading capacity was calculated as DL% = (DOX input—DOX recovery)/BPQDs-DOX@OPM × 100%.

### Construction of BPQDs-DOX@OPM combined drug delivery system

BPQDs-DOX@OPM was prepared by an extrusion method [29]. Appropriate amounts of OCM and PM were mixed in a 1:1 mass ratio. The OPM was prepared by ultrasonication in a water bath at 42 kHz and 100 W for 5 min followed by filtration through 400-nm and 200-nm porous membrane syringes 20 times successively. BPQDs-DOX was mixed with the OPM and filtered through 400-nm and 200-nm porous membrane syringes 20 times successively, and the excess OPM was removed by centrifugation at 2500 rpm for 10 min to obtain BPQDs-DOX@OPM.

### Investigation of the performance of OPM on BPQDs-DOX@OPM combined drug delivery system

SDS-PAGE gel electrophoresis and Western blot were used to detect the expressions of whole proteins and CD47 and EGFR signature proteins on BPQDs-DOX@OPM. These were used to verify the presence of OPM on BPQDs-DOX@OPM. RIPA lysate solution containing OCM, PM, or BPQDs-DOX@OPM protease inhibitor was added to lyse the cell membrane. After the sample was uploaded, electrophoresis was performed (160 V, 60 min), following which Coomassie Brilliant Blue staining was performed and photographed. Western blot was continued after the end of SDS-PAGE gel electrophoresis with membrane transfer operation (300 mA, 70 min). EGFR (1:1000) and CD47 (1:1000) primary antibodies were incubated, and secondary antibodies were incubated for 60 min and placed in a chemiluminescence detector to develop and take pictures.

### Characterization of BPQDs-DOX@OPM drug delivery system

The morphological structure of BPQDs-DOX@OPM was observed by transmission electron microscopy (TEM; JEOL JEM-F200, Japan), and the particle size was determined based on the microscopic morphology. Small amounts of BPQDs, OPM, BPQDs@OPM, and BPQDs-DOX@OPM were added to the carbon film copper net, and then the samples were dried, observed by TEM, and photographed. The surface charges were evaluated using Zetasizer Nano ZS (Malvern Zetasizer Nano ZS90, UK).

### Sustained drug release of BPQDs-DOX@OPM combined drug delivery system

BPQDs-DOX@OPM drug release was detected using a dialysis method. BPQDs-DOX@OPM irradiated with 808-nm NIR light for 10 min (1.0 W/cm^2^) at the beginning [BPQDs-DOX@OPM( +)]. Next, 2 mL each of BPQDs-DOX, BPQDs-DOX@OPM, and BPQDs-DOX@OPM ( +) were placed in individual dialysis bags, and the bags were placed in centrifuge tubes with a receiving medium volume of 10 mL. Then, the centrifuge tubes were placed in a thermostatic ultrasonic water bath (37 °C, 100 rpm) for 1, 2, 4, 8, 12, 16, 20, 24, 36, 48, 60, 24, 36, 48, 60, and 72 h. The optical density of DOX at 488 nm in the dialysate was measured by an enzyme standardizer, and the cumulative release was estimated from a standard curve of DOX concentration.

### Evaluation of BPQDs-DOX@OPM stability and photothermal performance

To assess the stability and the photothermal performance of BPQDs-DOX@OPM, 4 mL of BPQDs and BPQDs-DOX@OPM were exposed to the natural air environment. UV–Vis absorption spectra were recorded using a multifunctional enzyme marker at specific time points, namely 0, 2, 4, 6, and 8 days. The changes in absorbance values at 808 nm were statistically analyzed. Additionally, each solution group was subjected to near-infrared light irradiation (808 nm, 1.0 W/cm^2^) for 10 min on days 0, 2, 4, 6, and 8. During the irradiation, the BPQDs and BPQDs-DOX@OPM were allowed to stand, and the temperature variations were monitored using an infrared thermography camera. Images were captured every 30 s, and the corresponding temperatures were recorded. The results are summarized as follows.

### In vitro biocompatibility study of BPQDs@OPM drug delivery system

#### Cytotoxicity assay of BPQDs@OPM on Saos-2 cells

BPQDs@OPM with BPQD concentrations of 0.05, 0.1, 0.2, and 0.4 mg/mL were used, and PBS was used as the control group. After coincubation with Saos-2 cells for 24 h, the optical density of each well was detected at 450 nm in the enzyme standardizer. Using the same method, BPQDs@OPM with a BPQD concentration of 0.2 mg/mL was co-incubated with Saos-2 cells for 6, 12, 24, 48, and 72 h, and the optical density of each well was detected.

#### In vitro hemolysis rate study of BPQDs@OPM drug delivery system

BPQDs@OPM with BPQD concentrations of 0.05, 0.1, 0.2, and 0.4 mg/mL were used with normal saline (NS) as the negative control and distilled water as the positive control. From healthy Balb/c nude mice, 3 mL of whole blood was drawn and centrifuged to obtain erythrocytes, and a 20% erythrocyte suspension was prepared by adding physiological saline. Next, 100 μL of erythrocyte suspension was added to 900 μL of distilled water, NS, and different concentrations of BPQDs@OPM. After incubating for 2 h at 37 °C and centrifuging, pictures of the supernatant were taken, and the absorbance value of each well was measured at 540 nm on an enzyme standardizer. The hemolysis rate was calculated as (optical density of experimental group—optical density of negative control group)/(optical density of positive control group—optical density of negative control group).

### In vitro anti-tumor effect of BPQDs-DOX@OPM combined drug delivery system

#### Effect of BPQDs-DOX@OPM combined drug delivery system on the survival rate of Saos-2 cells

PBS, DOX, BPQDs, BPQDs@OPM, BPQDs-DOX, and BPQDs-DOX@OPM were treated with Saos-2 for 24 h. After adding drugs, each group was irradiated with 808-nm NIR light for 10 min (1 W/cm^2^). After 24 h, 10 μL of CCK-8 was added to each well. The optical density was detected at 450 nm by the enzyme standardizer, and the relative cell survival rate was calculated.

#### Effect of BPQDs-DOX@OPM combined drug delivery system on apoptosis of Saos-2 cells

The pro-apoptotic effect of the BPQDs-DOX@OPM combined drug delivery system on Saos-2 cells in vitro was detected by flow cytometry. Toward this end, 808-nm NIR light was irradiated on each well of PBS, DOX, BPQDs, BPQDs@OPM, BPQDs-DOX, and BPQDs-DOX@OPM groups, and the Saos-2 were treated for 24 h. The cells and corresponding supernatant were collected, the supernatant was discarded after centrifugation at 10,000×*g* for 5 min, and the cells were collected. Specifically, 5 × 10^3^ cells were collected, resuspended, and centrifuged at 1000×*g* for 5 min. Then, the supernatant was discarded, and the apoptosis rate was detected using a flow cytometer.

#### In vitro targeting study of BPQDs-DOX@OPM combined drug delivery system

The in vitro targeting of BPQDs-DOX@OPM was examined by confocal laser scanning microscopy (CLSM). DID fluorescently labeled BPQDs-DOX@OPM was produced [29] by mixing 200 µL of a PM and OCM mixture with 1:1 weight ratio of proteins with 15 µL of DiD for 2 h. Then, the membrane solution was mixed with an appropriate amount of BPQDs-DOX. Logarithmic growth stage Saos-2, RAW264.7, HBMEC, and 3T3 were inoculated in 24-well plates and cultured for 24 h after cell apposition. DID-(BPQDs-DOX@OPM) was added to each well, after which we incubated without light for 4 h. The sample was imaged using CLSM with a laser excitation wavelength of 644 nm and emission wavelength of 665 nm.

### In vivo targeting study of BPQDs-DOX@OPM combined drug delivery system

#### Small animal in vivo optical imaging targeting assay

A small animal live imaging system (Xenogen IVIS Lumina XRMS) was used to assess the distribution of BPQDs-DOX@OPM in animals. Regarding the floral cyanine dye cy5 fluorescent labeling scheme [30], cy5-BPQDs were constructed by mixing appropriate amounts of BPQDs with cy5 and then mixing the resulting cy5-BPQDs with DOX and OPM to obtain cy5-(BPQDs-DOX) and cy5-(BPQDs-DOX@OPM), respectively, according to the extrusion method described above. After the experimental nude mice were randomly grouped, each treatment group was injected with 200 μL of saline, cy5-(BPQDs-DOX), and cy5-(BPQDs-DOX@OPM) successively (cy5 injection dose of 5 μg/kg in the cy5-containing drug). The fluorescence intensity and distribution were measured 6, 12, 24, and 48 h after tail vein injection using the Xenogen IVIS lumina XR imaging system. After 48 h, all mice were euthanized, and their tumors and major organs were collected. The fluorescence intensity and distribution were then measured again using the same imaging system.

#### DOX aggregation in tumor tissues

Fluorescence microscopy was performed to detect DOX aggregation in the tumor tissue. To verify the targeting ability of the BPQDs-DOX@OPM combined drug delivery system, we observed the aggregation of DOX within the tumor in different administration groups; DOX is self-fluorescent in red because it contains an anthracycline chromophore at the center [31]. At the end of the small animal in vivo optical imaging experiments, tumor tissues from each treatment group were collected, placed in a composite medium, and rapidly frozen in liquid nitrogen. After tissue fixation, sections (10 μm) were prepared using a thermostatic frozen section machine at − 20 °C. Fluorescence microscopy was used to observe intratumoral DOX aggregation in each treatment group, with an excitation wavelength of 480 nm and an emission wavelength of 593 nm.

### In vivo temperature rise curve of BPQDs-DOX@OPM combined drug delivery system

Three groups were established to replicate the in vitro photothermal rise curve: NS, BPQDs, and BPQDs-DOX@OPM. Tumor-bearing Balb/c nude mice were used in the experiment. Each group received a tail vein injection of 200 μL of the respective drug. The temperature changes at the tumor site were monitored using an infrared thermal imager, with pictures taken every 30 s. The infrared thermal imager was also used to measure the temperature of the tumor site in each group of animals.

### In vivo anti-tumor effect of BPQDs-DOX@OPM combined drug delivery system

The in vivo anti-tumor effect of the BPQDs-DOX@OPM combined drug delivery system was evaluated based on the tumor tissue volume growth during treatment. OS animals with tumors and good health were randomly grouped (NS, BPQDs, DOX, BPQDs-@OPM, BPQDs-DOX, and BPQDs-DOX@OPM) and injected with 0.2 mL of drug in the tail vein. The tumor site was irradiated with 808 nm NIR light 24 and 48 h after injection in each group. To assess the anti-tumor efficacy, we measured the tumor volume using the formula V = π/6 × L × W^2^, where L represents the longest diameter of the tumor, and W represents one of the longest transverse diameters perpendicular to the longest diameter in millimeters cubed. Additionally, we measured the animal weight on alternate days. Finally, the mice were euthanized after the treatment, and tumor tissues were collected for further analysis. The tumor tissues were stained with H&E to evaluate the pathological damage caused by each treatment group and determine the efficacy of the treatments.

### In vivo biosafety assessment of BPQDs-DOX@OPM combined drug delivery system

The in vivo biosafety of BPQDs-DOX@OPM was assessed based on changes in animal body weight and immunohistochemical H&E staining. After treatment, the major organs of the mice were dissected and sampled. After embedding in paraffin, the sections were stained with hematoxylin for 30 s and 0.5% eosin for 30 s. The sections were sealed and observed under a microscope.

### Data analysis

In this study, all data were presented as mean ± standard deviation. The GraphPad Prism 9.0 software was used for data analysis. One-way analysis of variance (ANOVA) was conducted, followed by Tukey’s post-hoc comparison tests for statistical analysis. Ns indicated no significance. Statistically significant differences were indicated by *P < 0.05, **P < 0.01, and ***P < 0.005.

## Results

### Examination of OPM properties on BPQDs-DOX@OPM

**Fig. 2:**
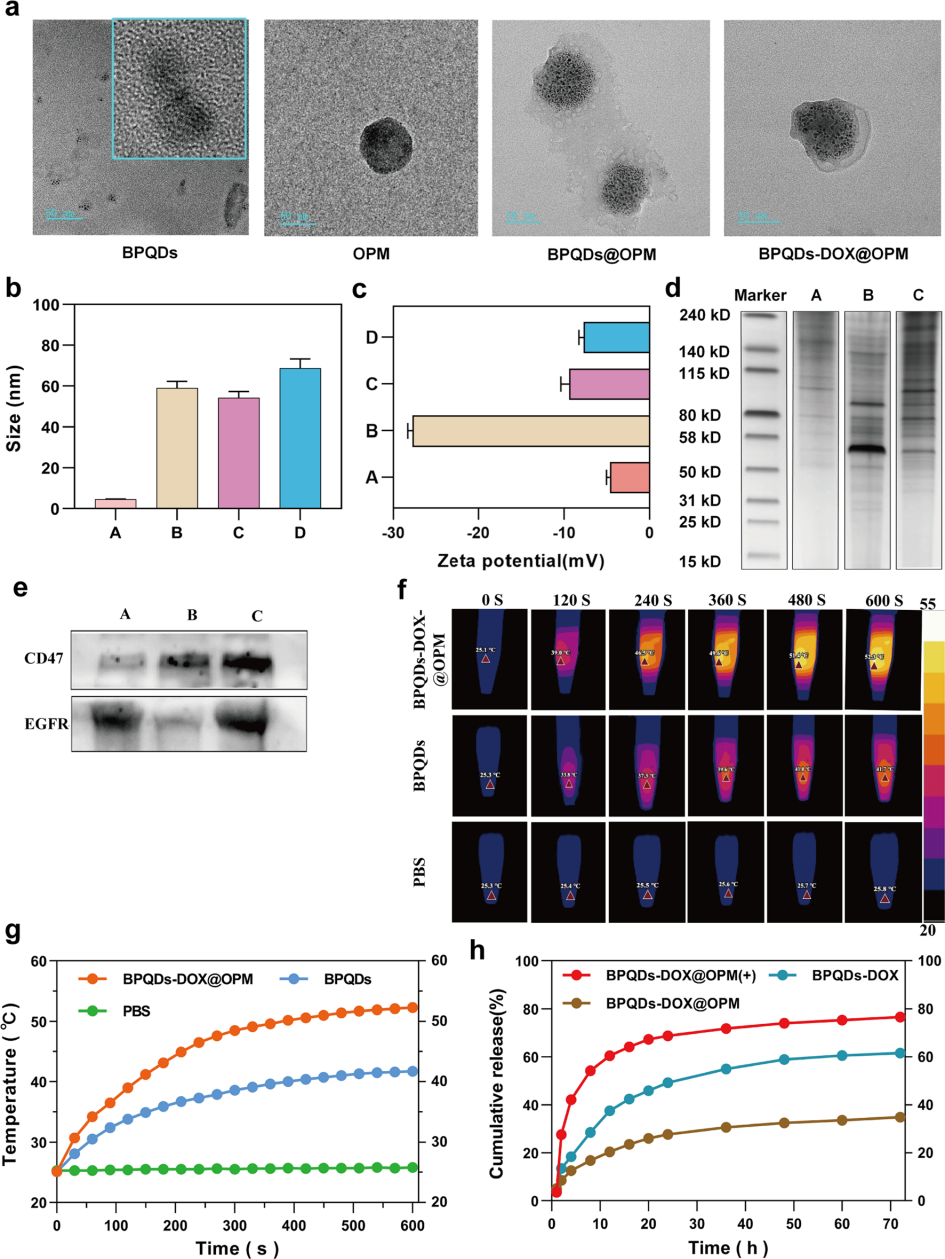
BPQDs-DOX@OPM characterization. **a** Transmission electron micrographs of BPQDs, OPM, BPQDs@OPM, and BPQDs-DOX@OPM (size: 50 nm). **b**, **c** Particle size and zeta potential of each group (A: BPQDs, B: OPM, C: BPQDs@OPM, D: BPQDs-DOX@OPM). **d** SDS-PAGE gel electrophoresis result (A: OCM, B: PM, C: BPQDs-DOX@OPM). **e** Western blot analysis result (A: OCM, B: PM, C: BPQDs-DOX@OPM). **f**, **g** Photothermal performance and temperature rise curve of BPQDs-DOX@OPM. **h** DOX release characteristics of BPQDs-DOX@OPM

The cellular hybrid membrane-encapsulated nanoparticles consist of two main parts: nanocarrier core and surface cell membrane shell structure [32]. Because the hybrid membrane can retain glycoproteins, lipids, and proteins on the entire cell membrane surface, it can endow the nanoparticles with functions and properties inherent to the source cell. For example, CD47 on the platelet surface can interact with immune cells to inhibit immune clearance [27], and cancer cells can be homotypically targeted [28]. To examine the proteins on BPQDs-DOX@OPM, we examined the hybrid membrane whole protein and signature protein expression by SDS-PAGE gel electrophoresis and Western blot, respectively. The SDS-PAGE gel electrophoresis results are shown in Fig. 2d; they indicate that the BPQDs-DOX@OPM group has both OCM and PM on the protein. The Western blot results are shown in Fig. 2e; they indicate that the OCM, PM, and BPQDs-DOX@OPM groups showed the expression of the signature protein EGFR, signature protein CD47, and both EGFR and CD47, respectively. The results of SDS- PAGE gel electrophoresis and Western blot indicated that BPQDs-DOX@OPM contained an OPM heterodimeric membrane with full protein expression.

### BPQDs-DOX@OPM-like characteristics

In cell membrane-mimetic encapsulation of small-sized nanocarriers, the size of the cell membrane-modified nanocarriers is usually close to that of the cell membrane, and the surface potential of the drug delivery system is close to that of the cell membrane [33, 34]. The TEM results (Fig. 2a) showed that the BPQDs as well as BPQDs@OPM hybrid membrane vesicles had a rounded morphology and good dispersion, and OPM had a shell structure that completely encapsulated multiple BPQDs. The TEM results (Fig. 2b) showed that the particle sizes of the BPQDs, OPM, BPQDs@OPM, and BPQDs-DOX@OPM groups were 4.53 ± 1.36 nm, 58.96 ± 13.65 nm, 54.22 ± 17.69 nm, and 68.80 ± 23.78 nm, respectively; the particle size of BPQDs-DOX@OPM was close to that of OPM vesicles. The zeta potentials (Fig. 2c) of BPQDs, OPM, BPQDs@OPM, and BPQDs-DOX@OPM as measured by DLS were − 18.07 ± 0.46 mv, − 9.43 ± 0.78 mv, − 4.64 ± 0.34 mv, and − 7.70 ± 0.46 mv, respectively; the zeta potential of BPQDs-DOX@OPM was close to that of OPM.

### Photothermal conversion performance of BPQDs-DOX@OPM

Zero-dimensional BPQDs with a 0–10 nm size exhibit a wide absorption range throughout the visible region. Consequently, they have good photothermal conversion efficiency [35]. However, BPQDs are susceptible to oxidative degradation in an environment containing oxygen and water; biofilm encapsulation can isolate the internal BPQDs from oxygen and water to improve their stability [36]. As shown in Fig. 2f and g, After exposing the BPQDs and BPQDs-DOX@OPM to the air environment for 4 days, each set of solutions was irradiated with near-infrared light (808 nm, 1.0 W/cm^2^) for 10 min. The temperature of the PBS group did not change significantly. By contrast, temperatures of the BPQDs and BPQDs-DOX@OPM groups increased rapidly and reached 41.7 °C and 52.3 °C, respectively, after 600 s. Further, the final temperature of the BPQDs-DOX@OPM was higher than both the BPQDs and the tissue cell lethal temperature of 48 °C [11].

### BPQDs-DOX@OPM drug slow release curve

The calculated drug loading and encapsulation efficiency were 36.2% and 73.4%, respectively, based on the linear regression equation of the DOX standard curve. As shown in Fig. 2h, the 72-h release of DOX from BPQDs-DOX@OPM and BPQDs-DOX was 34.88% and 61.53%, respectively; this indicated that the OPM hybrid film encapsulation system could slow down the release of DOX and help prolong the half-life of BPQDs-DOX@OPM in the blood circulation system. After irradiation with 808-nm NIR light, owing to photostimulation-responsive release, the 72-h DOX release of the BPQDs-DOX@OPM(+) group was 76.04%; this was significantly higher than that of the BPQDs-DOX@OPM group without light irradiation.

### In vitro stability assessment of BPQDs-DOX@OPM

Zero-dimensional BPQDs, with sizes ranging from 0 to 10 nm, demonstrate broad absorption across the visible region, indicating favorable photothermal conversion efficiency. However, BPQDs are susceptible to oxidative degradation in the presence of oxygen and water. Biofilm encapsulation technology was employed to isolate the internal BPQDs from oxygen and water to enhance their stability. To evaluate the enhanced stability conferred by OPM encapsulation, BPQDs and BPQDs-DOX@OPM, both with consistent concentrations, were dispersed in water and exposed to air for 8 days. Optical property measurements were conducted at specific intervals (0, 2, 4, 6, and 8 days).

**Fig. 3:**
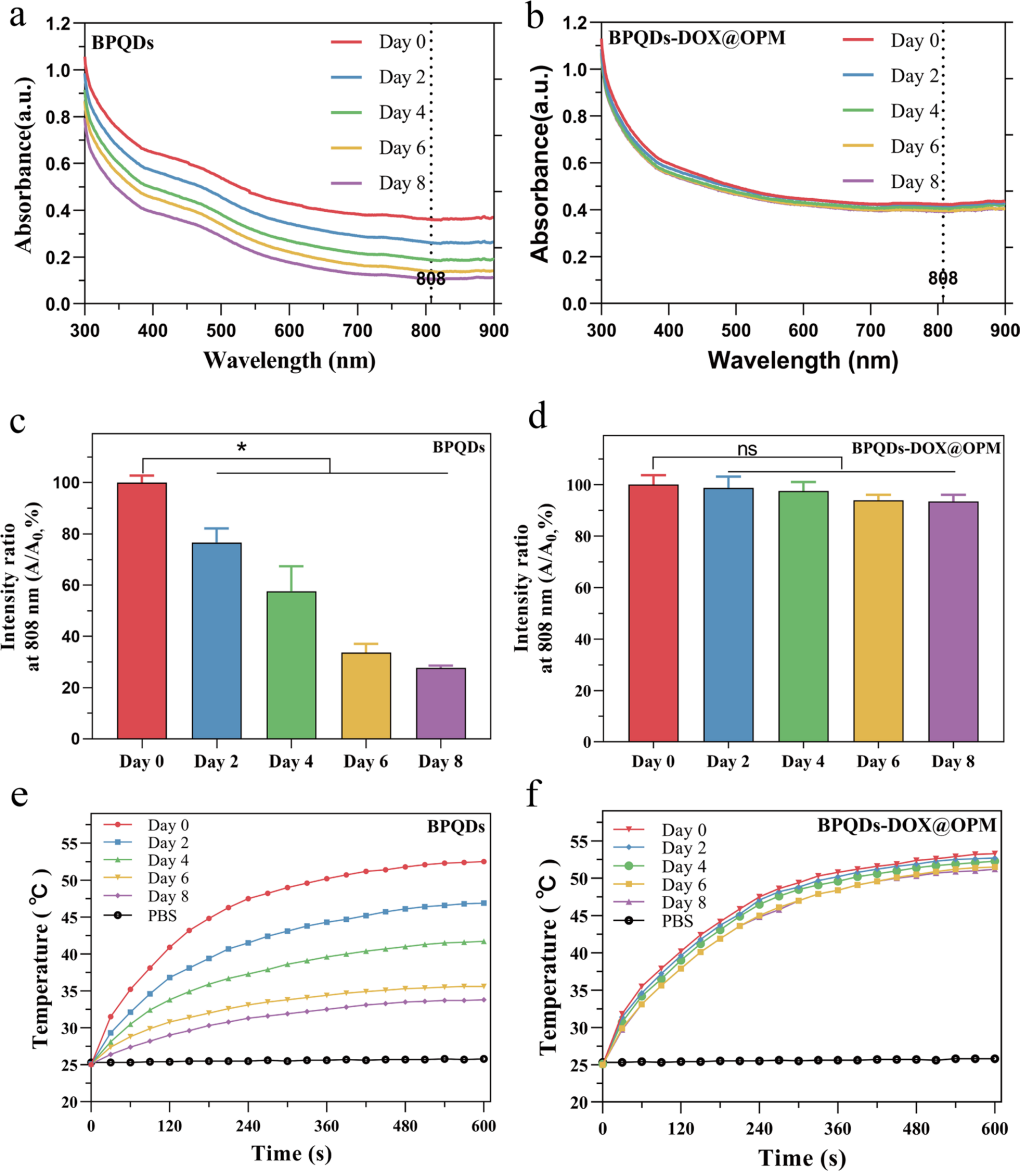
UV–Vis absorption spectra of BPQDs and BPQDs-DOX@OPM at different time points and the corresponding temperature rise curve. **a** Absorption spectra of BPQDs at 0, 2, 4, 6, and 8 days; **b** absorption spectra of BPQDs-DOX@OPM at 0, 2, 4, 6, and 8 days; **c** statistical analysis of the absorbance of BPQDs at 808 nm at different time points; **d** statistical analysis of the absorbance of BPQDs-DOX@OPM at 808 nm at different time points; **e** temperature rise curves of BPQDs on days 0, 2, 4, 6, and 8 days; **f** temperature rise curves of BPQDs-DOX@OPM on days 0, 2, 4, 6, and 8 days

Figure 3a, b illustrates the typical broad absorption bands in the UV and NIR regions exhibited by both BPQDs and BPQDs-DOX@OPM. However, the absorbance intensity of BPQDs in water decreased over time, whereas no significant decrease in absorbance intensity was observed for BPQDs-DOX@OPM. The percentage change in absorbance intensity at 808 nm for BPQDs and BPQDs-DOX@OPM is depicted in Fig. 3c, d, respectively. After 2 days, the absorbance intensity of BPQDs decreased by 23.4% ± 4.47%, and after 8 days, it decreased by 72.3% ± 0.71%. This decrease was attributed to the degradation of bare BPQDs, resulting in diminished absorbance. In contrast, the absorbance of BPQDs-DOX@OPM remained more stable, with only a 6.57% ± 2.14% decrease after 8 days. This enhanced stability can be attributed to the shell structure of OPM, which reduces the influence of oxygen and water on BPQDs, thereby improving the stability of BPQDs-DOX@OPM under environmental conditions.

Figure 3e presents the temperature rise curve of BPQDs, showing a decrease over time due to the unstable nature and easy oxidative degradation of BPQDs. Conversely, Fig. 3f demonstrates that the temperature rise curve of BPQDs-DOX@OPM does not exhibit a significant decrease over time. This observation aligns with the results obtained from the UV–vis absorption spectra of BPQDs-DOX@OPM, confirming that the OPM shell structure enhances the stability of BPQDs under environmental conditions.

### In vitro biocompatibility of BPQDs@OPM drug delivery system

#### Cytotoxicity of BPQDs@OPM drug delivery system on Saos-2 cells

**Fig. 4:**
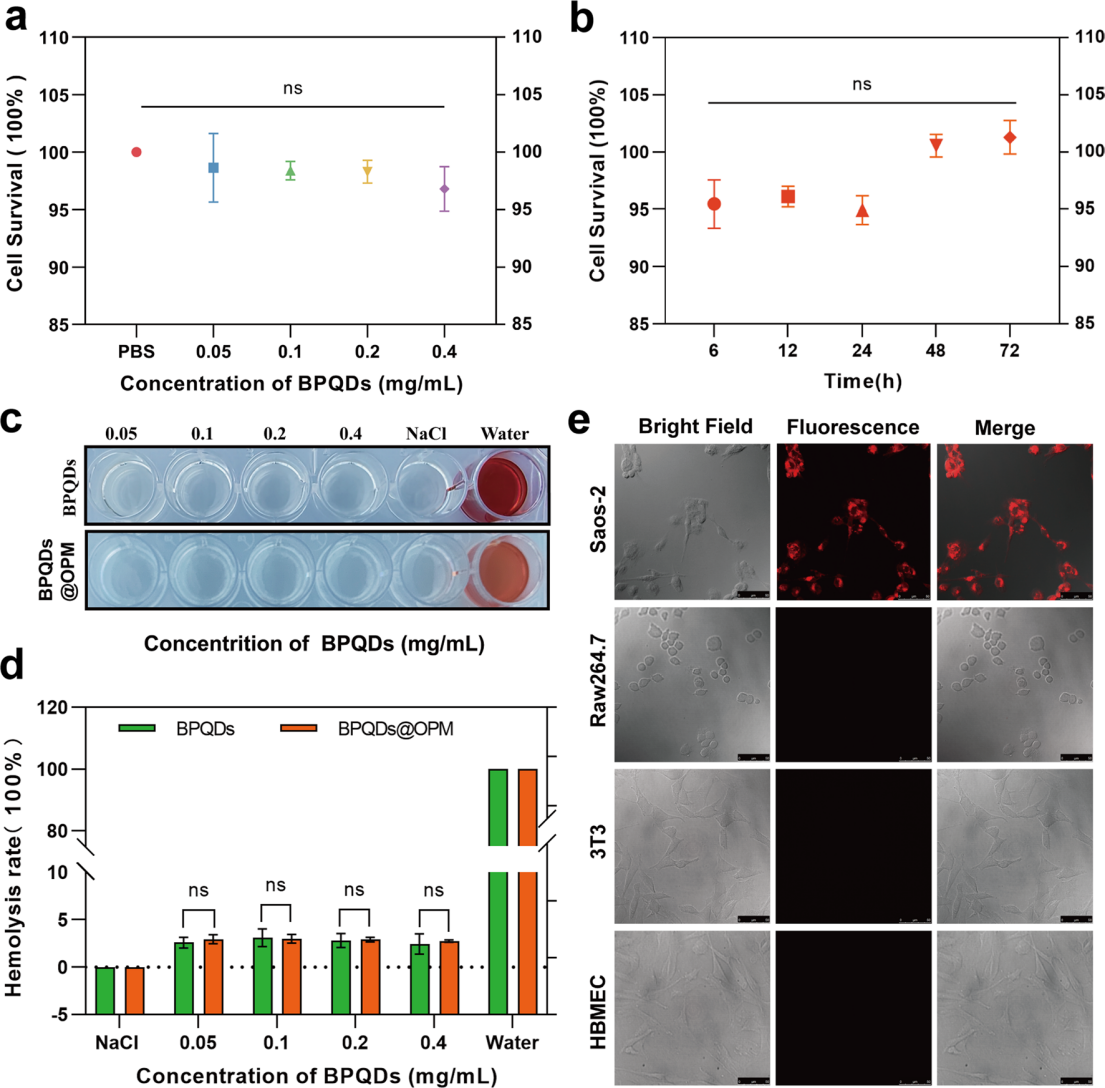
BPQDs-DOX@OPM in vitro performance study. **a** Cell viability of BPQDs@OPM with different BPQD concentrations after coincubation with Saos-2 for 24 h. **b** Cell viability of BPQDs@OPM with BPQD concentration of 0.2 mg/mL after coincubation with Saos-2 cells for different periods. **c** Photographs of supernatants of BPQDs and BPQDs-DOX@OPM hemolysis experiments. **d** Calculation of hemolysis rates of BPQDs and BPQDs-DOX@OPM. **e** DID fluorescence-labeled BPQDs-DOX@OPM after coincubation with Saos-2, 3T3, and HBMEC, and RAW264.7 for 4 h. Laser confocal microscopy imaging (size: 50 μm)

The in vitro biocompatibility assessment of nanocarriers regarding tissue cytotoxicity and hemocompatibility is considered the most reliable and simplest technical tool in biosafety assessment [37]. Studying the dose- and time-dependent cytotoxicity of nanocarriers is one of the most common methods to detect cytotoxicity in vitro [38]. Therefore, as shown in Fig. 4a, Saos-2 was plated for 12 h after BPQDs@OPM treated cells. The results of the CCK-8 cell proliferation activity assay showed that the relative cell survival rate of each group was maintained above 95% after coincubation of BPQDs@OPM with cells at different concentrations for 24 h, and the difference was not statistically significant. As shown in Fig. 4b, after 12 h of Saos-2 cell plating, the BPQDs@OPM system was coincubated with Saos-2 cells for 6, 12, 24, 48, and 72 h, and the CCK-8 cells were assayed for relative cell viability. The assay results showed that the cell proliferation activity of the BPQDs@OPM drug delivery system was maintained above 95% at different periods of coincubation with Saos-2 cells, and the differences were not statistically different. The dose- and time-effect relationships of BPQDs@OPM both indicated its high biocompatibility.

#### Hemocompatibility of BPQDs@OPM drug delivery system

Chemotherapeutic drug carriers are mostly delivered to the tumor site through blood circulation. Investigating their hemocompatibility can help further understand the safety and efficacy of drug delivery systems [39, 40]. Therefore, the erythrocyte hemolysis assay was chosen to verify the BPQDs@OPM hemocompatibility in this study. As shown in Fig. 4c and d, after coincubation of each treatment group with the erythrocyte suspension for 1 h, the erythrocyte hemolysis rates of BPQDs@OPM groups with BPQD concentrations of 0.05, 0.1, 0.2, and 0.4 mg/mL were below 3%. The BPQDs group with 0.1 mg/mL BPQD concentration showed the highest erythrocyte hemolysis rate of 3.10 ± 0.75%. The hemolysis rate of BPQDs and BPQDs-DOX in all concentration groups was less than 5%, in accordance with the required hemolysis rate for biological materials; this indicated that both BPQDs and BPQDs@OPM had good hemocompatibility.

### In vitro targeting study of BPQDs-DOX@OPM

As shown in Fig. 4e, surface antigens are reportedly responsible for the homotypic adhesion of cancer cells, and the cancer cell membrane camouflage platform exhibits ideal homotypic cancer-targeting ability [41]. CD47 on the platelet surface can signal “do not eat me” to phagocytes, thus gaining immune evasion ability and conferring a longer half-life to the drug delivery system [42]. To verify the isotype targeting ability and immune evasion capacity of BPQDs-DOX@OPM, the cellular uptake of DiD-labeled BPQDs-DOX@OPM was evaluated using Saos-2, 3T3, HBMEC, and RAW264.7 as controls. DID-(BPQDs-DOX@OPM) exhibited strong red fluorescence after coincubation with Saos-2 cells for 4 h. No significant fluorescence distribution was seen in 3T3, HBMEC, and RAW264.7 cells, indicating that BPQDs-DOX@OPM has OS cell isotype targeting and immune evasion abilities.

### In vitro anti-tumor effect study of BPQDs-DOX@OPM

#### Effect of BPQDs-DOX@OPM on proliferative activity of Saos-2 cells

**Fig. 5:**
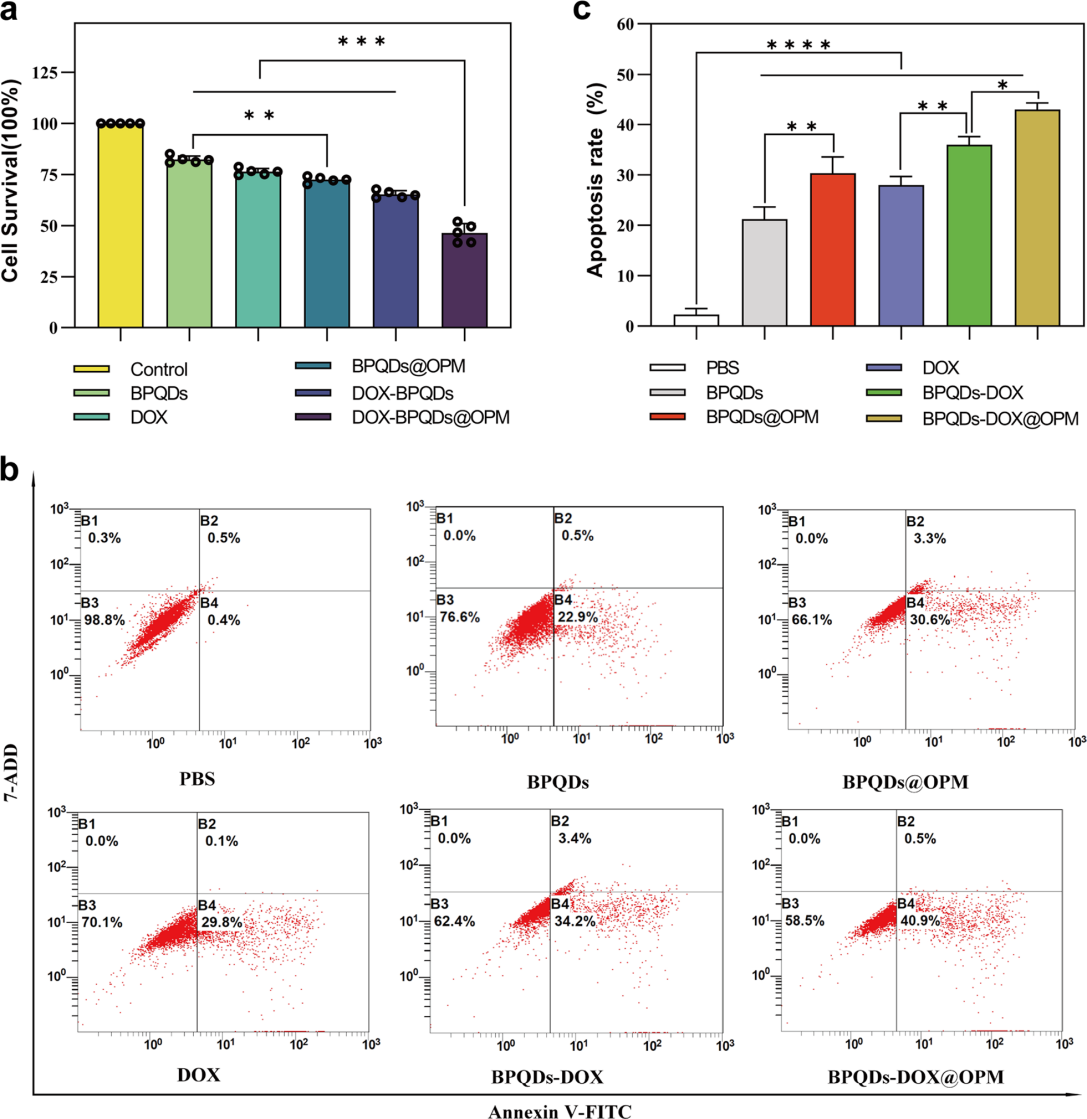
BPQDs-DOX@OPM in vitro efficacy study. **a** Each treatment group treated with Saos-2 cells for 24 h, relative cell survival rate. **b** Apoptosis rate of Saos-2 cells treated with each treatment group for 24 h. **c** Statistical analysis of apoptosis rate of Saos-2 cells. (*P < 0.05,**P < 0.01, ****P < 0.001)

Necrosis and apoptosis are the most common forms of cell death. To investigate the in vitro anti-tumor effect of the BPQDs-DOX@OPM combined drug delivery system, we detected the proliferative activity of Saos-2 by CCK-8 and the apoptosis rate by flow cytometry. As shown in Fig. 5a, different groups were set (i.e., Control, BPQDs, BPQDs@OPM, DOX, BPQDs-DOX, and BPQDs-DOX@OPM), and each group was irradiated with 808-nm NIR light when adding the drug. After treating Saos-2 cells for 24 h, the cell survival rates of the BPQDs and BPQDs@OPM were 82.32% ± 1.54% and 72.48% ± 1.30%, respectively. The cell survival rate in the BPQDs@OPM group was lower than that in the BPQDs group, indicating that the surface modification of the OPM could enhance the stability of BPQDs. BPQDs-DOX@OPM was significantly lower, indicating that the combined treatment had a more potent killing effect on OS.

#### Effect of BPQDs-DOX@OPM combined drug delivery system on apoptosis of Saos-2 cells

As shown in Fig. 5b, c, different groups (i.e., Control, BPQDs, BPQDs@OPM, DOX, BPQDs-DOX, and BPQDs-DOX@OPM) were set up to treat Saos-2 cells for 24 h. Each group was irradiated with 808 nm NIR light when the drug was added. Compared with the apoptosis rate of the control group, those of the DOX, BPQDs@OPM, and BPQDs-DOX groups were 28.00 ± 1.38%, 30.36 ± 2.64%, and 36 ± 1.35%, respectively. The BPQDs-DOX@OPM combined treatment group had an apoptosis rate of 42.97 ± 1.13%, indicating that the combined therapy had a more powerful killing effect on OS than single-agent therapy.

### In vivo targeting study of BPQDs-DOX@OPM

#### In vivo targeting assay with in vivo optical imaging of small animals

**Fig. 6:**
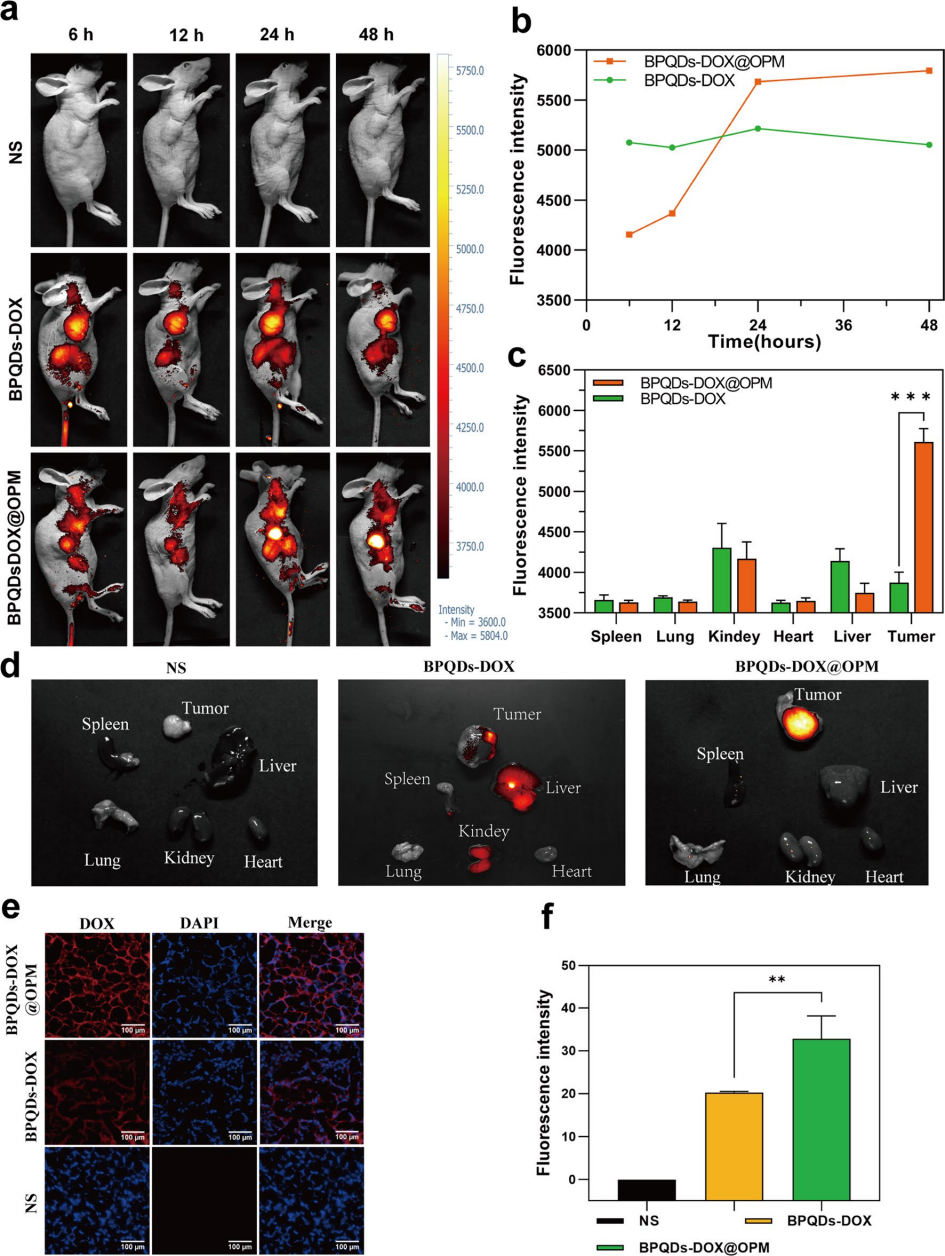
BPQDs-DOX@OPM in vivo targeting study. **a**, **b** Fluorescence intensity and distribution in animals after tail vein injection of cy5-labeled BPQDs-DOX@OPM at 6, 12, 24, and 48 h. **c**, **d** Fluorescence distribution of tumor tissues and heart, liver, spleen, lungs, and kidneys of animals 48 h after tail vein injection of cy5-labeled BPQDs-DOX@OPM. **e** Fluorescence distribution of DOX in animal tumor tissues 48 h after tail vein injection of cy5-labeled BPQDs-DOX@OPM. **f** Analysis of DOX fluorescence intensity in animal tumor tissues

To further confirm the hypothesis of targeting, the BPQDs-DOX@OPM combination drug delivery system was labeled with the fluorescent dye Cy5, and its in vivo biodistribution was detected by in vivo fluorescence imaging of small animals. Figure 6a, b shows that BPQD-DOX partially accumulated in tumor tissues at 6 and 12 h after drug injection compared with the NS group. This is due to the “passive targeting” effect [43], and the aggregation of BPQDs-DOX in tumor tissues and major organs decreased at 48 h, probably owing to its smaller size and rapid metabolic clearance by the body. Compared with BPQDs-DOX, BPQDs-DOX@OPM showed significant aggregation in tumor tissues 24 h after intravenous injection, and its aggregation in tumor tissues did not show a significant decrease after 48 h. As shown in Fig. 6c, d, tumors and major organs (i.e., heart, liver, spleen, lungs, and kidney) were collected 48 h after drug treatment. The fluorescence imaging results showed that no obvious fluorescence distribution was observed in all major organs of the BPQDs-DOX-treated group, and the fluorescence intensity in the tumor tissues was low, indicating that BPQDs-DOX was rapidly cleared by the organism at 48 h. The BPQDs-DOX@OPM-treated group showed an obvious fluorescence distribution in the tumor tissue but not in the main organs. This indicated that BPQDs-DOX@OPM has good in vivo targeting and long circulation time.

#### DOX aggregation in tumor tissues

As shown in Fig. 6e and f, 48 h after OS mice were treated with NS, BPQDs-DOX- and BPQDs-DOX@OPM, the tumor tissues were taken to make frozen sections, and the sections were restained with DAPI and observed under a fluorescence microscope. The bright fluorescence of DOX was observed in the BPQDs-DOX@OPM group, and the fluorescence intensity was significantly stronger than that of the BPQDs-DOX group; this confirmed the excellent targeted drug delivery performance of BPQDs-DOX@OPM.

#### In vivo temperature rise curve of BPQDs-DOX@OPM

**Fig. 7:**
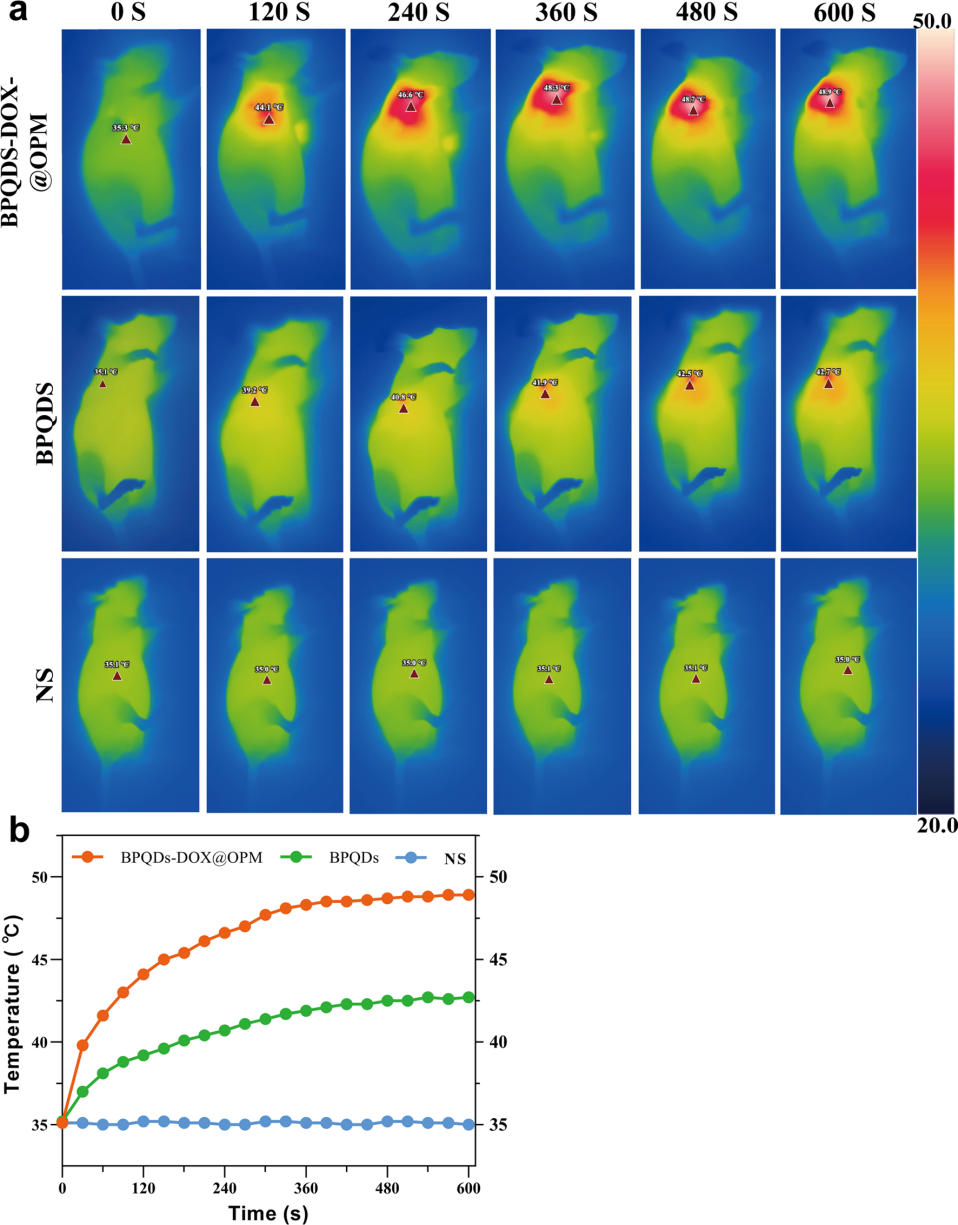
BPQDs-DOX@OPM in vivo temperature rise curves. **a** NS, BPQDs, BPQDs-DOX@OPM In vivo thermal imaging photography; **b** NS, BPQDs, BPQDs-DOX@OPM In vivo temperature rise curves

To further investigate the in vivo photothermal performance of the BPQDs-DOX@OPM co-loading system, three groups were established: NS saline group, BPQDs group, and BPQDs-DOX@OPM group. Figure 7a, b depicts that each group was subjected to 808 nm near-infrared light irradiation for 10 min. The NS group showed no significant temperature change, while the BPQDs and BPQDs-DOX@OPM groups exhibited a rapid temperature increase during irradiation. After 10 min, the BPQDs and BPQDs-DOX@OPM groups reached 42.7 °C and 48.9 °C, respectively. The final temperature of the BPQDs-DOX@OPM group was higher than that of the BPQDs group, surpassing the lethal temperature of tissue cells at 42 °C. This observation indicates that BPQDs-DOX@OPM demonstrated excellent in vivo photothermal performance. The temperature elevation in the tumor area of the BPQDs-DOX@OPM group can be attributed to the combined effect of effective tumor targeting and the high stability of BPQDs.

### In vivo anti-tumor effect of BPQDs-DOX@OPM combined drug delivery system

**Fig. 8:**
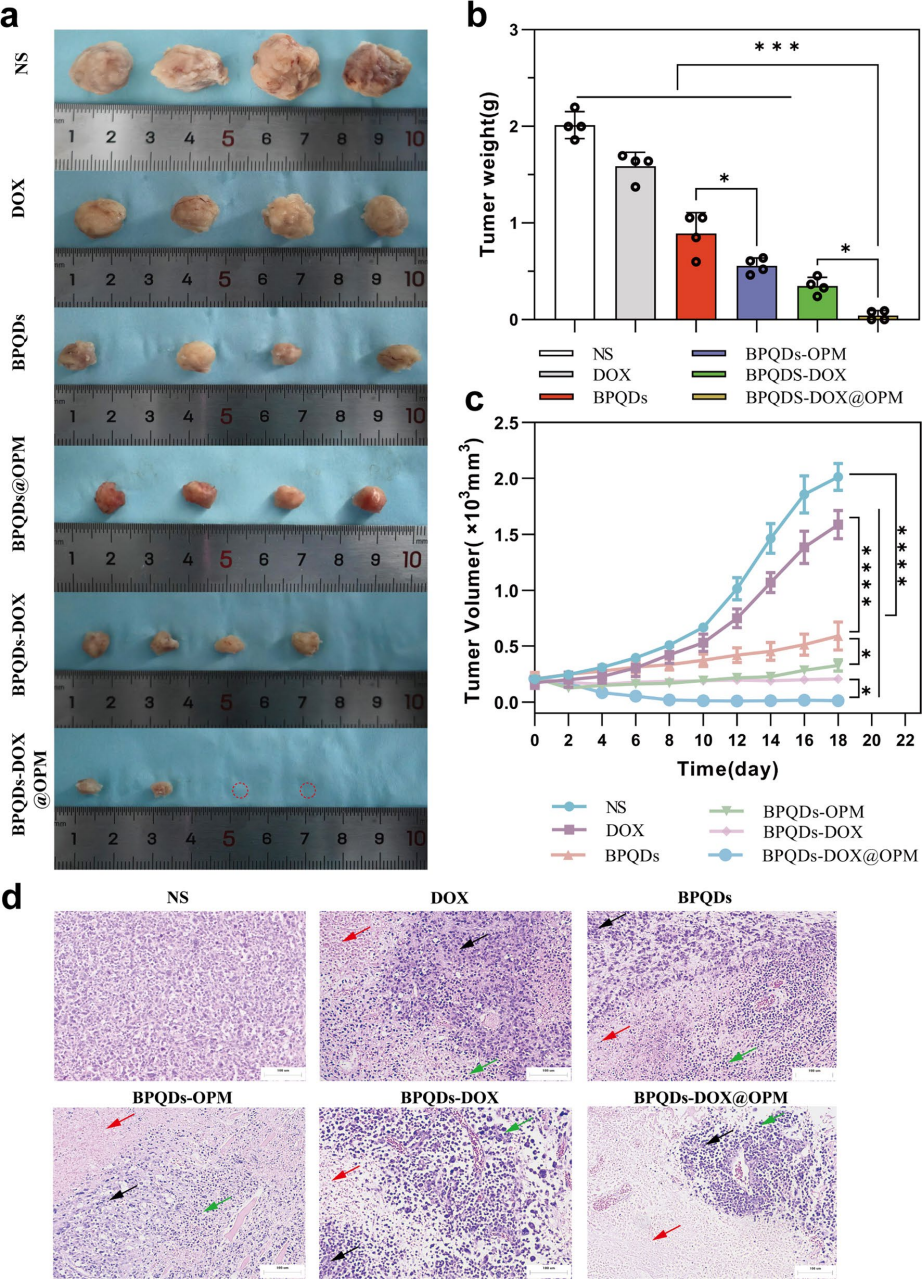
Anti-tumor effects of BPQDs-DOX@OPM in vivo. **a** Tumor tissues after 18 days of intravenous injection of NS, BPQDs, DOX, BPQDs-@OPM, BPQDs-DOX, and BPQDs-DOX@OPM. **b** Measured tumor tissue weights after 18 days of treatment with NS, BPQDs, DOX, BPQDs-@OPM, BPQDs-DOX, and BPQDs-DOX@OPM. **c** Tumor volume change curves during NS, BPQDs, DOX, BPQDs-@OPM, BPQDs-DOX, and BPQDs-DOX@OPM treatment. **d** H&E stained images of tumor (size: 100 μm; Black arrows: aggregated tumor cell growth; Red arrows: tumor tissue necrosis; Green arrows: inflammatory cell infiltration.)

As shown in Fig. 8a–c, the in vivo therapeutic effects of different treatment groups on OS were obviously different. Compared with the NS group, the BPQDs group and BPQDs@OPM group significantly reduced the rate of tumor volume increase. This is due to the good therapeutic effect of PTT on local tumor growth. The tumor volume in the BPQDs@OPM group was significantly smaller (p < 0.05) than in the BPQDs group. This was because the surface modification of OPM significantly improved the stability and slowed down the oxidative degradation of BPQDs; this was consistent with our preliminary assumption. The tumor growth rate inhibition was more evident in the BPQDs-DOX group than in the BPQDs and DOX groups, indicating that combined chemotherapy with PTT has more obvious advantages than a single-agent therapy for tumor treatment. However, BPQDs-DOX may be cleared by the immune system and renal excretory system in vivo owing to the immune clearance system and small size. In addition, BPQDs undergo further degradation upon exposure to oxygen and water, leading to reduced efficacy. Our BPQDs-DOX@OPM combined treatment group showed the most pronounced tumor growth inhibition at the end of the treatment.

### H&E staining of tumor tissue

Figure 8d illustrates the H&E staining of tumor tissues after the treatment. In the NS group, tumor cells did not exhibit significant necrosis and displayed varying cell sizes, noticeable nuclear heterogeneity, and disordered arrangement. In contrast, the remaining treatment groups exhibited tumor necrosis, intensified nuclear staining, and infiltration of inflammatory cells. Notably, the BPQDs-DOX@OPM group demonstrated extensive tumor cell necrosis, consolidation of nuclei with deepened staining, and sparse arrangement of tumor cells without nuclear schizogony. Moreover, in the BPQDs-DOX@OPM group, there was widespread tumor cell necrosis, deepening of nuclear solidification staining, and infiltration of inflammatory cells in the residual tumor cells.

### In vivo biosafety studies of BPQDs-DOX@OPM combined drug delivery system

**Fig. 9:**
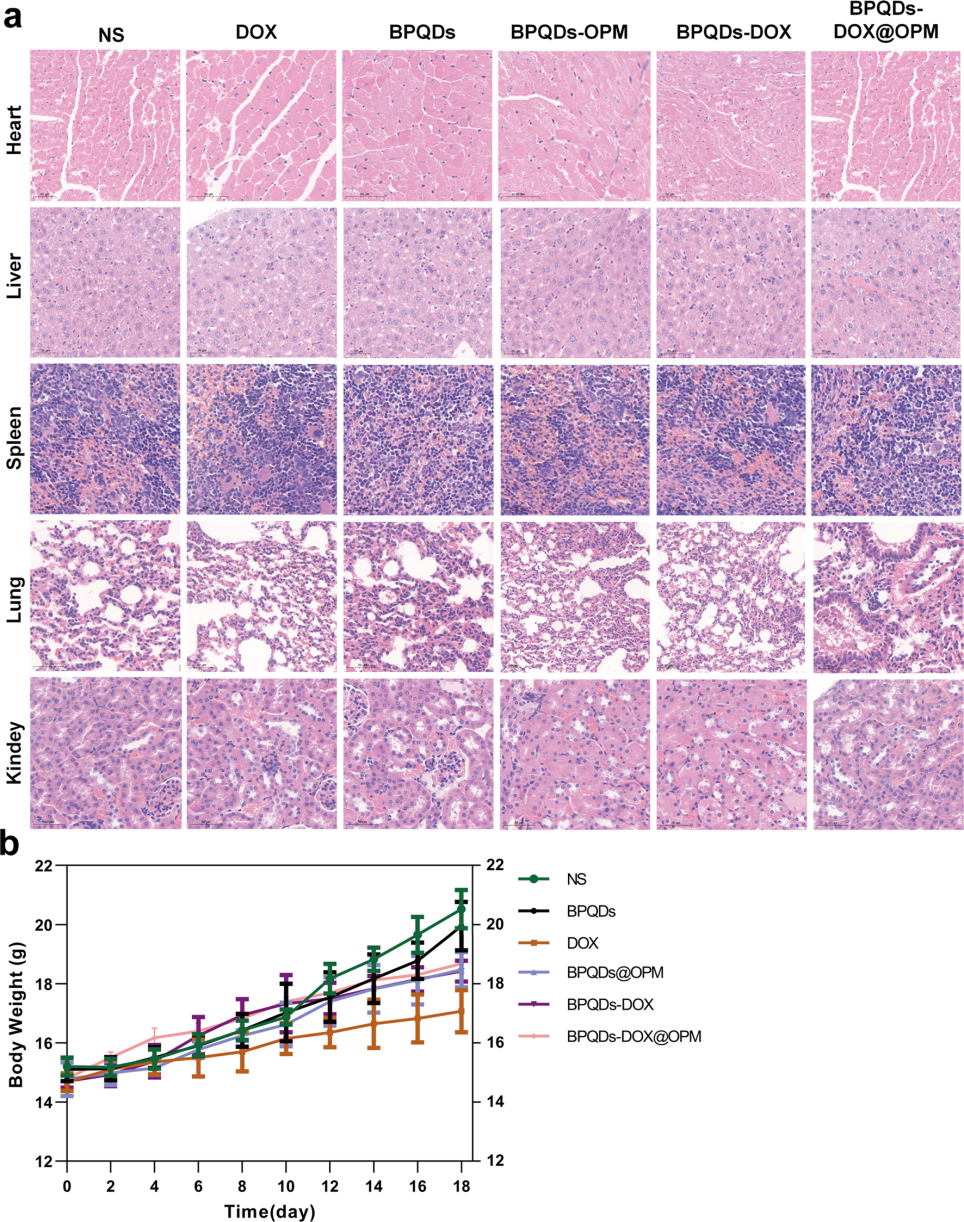
In vivo biosafety assessment of BPQDs-DOX@OPM. **a** H&E stained sections of each major organ of animals 18 days after intravenous injection of NS, BPQDs, DOX, BPQDs-@OPM, BPQDs-DOX, and BPQDs-DOX@OPM. **b** Body weight changes of animals in each group during NS, BPQDs, DOX, BPQDs-@OPM, BPQDs-DOX, and BPQDs-DOX@OPM treatment. (size: 100 μm)

The toxic effects of nano-drug delivery systems on major organs and the whole system are considered an essential direction for assessing drug toxicity, and one of their leading indicators is a change in body weight [29]. As shown in Fig. 9a, the trend of body weight change in mice was recorded during treatment, and no significant decrease in body weight was observed in the BPQDs-DOX@OPM and BPQDs@OPM treatment groups. After the end of BPQDs-DOX@OPM in vivo treatment, the heart, liver, spleen, lungs, and kidneys of the sampled mice were dissected. As shown in Fig. 9b, the H&E staining results indicated that the BPQDs-DOX@ OPM group showed no significant damage to all organs, indicating that the BPQDs-DOX@OPM combined drug delivery system had high biosafety.

## Discussion

In this study, we developed a BPQDs-DOX@OPM combined drug delivery system for the treatment of OS. The OPM serves as a protective shell that encapsulates multiple BPQDs-DOX and controls the release rate of DOX. NIR light irradiation is employed to accelerate the release of DOX, resulting in a prolonged circulation time and photostimulation-responsive release of BPQDs-DOX@OPM. The OPM encapsulation system also enhances the stability of BPQDs by minimizing their exposure to oxygen and water, thereby improving their photothermal therapeutic effects.

The in vitro and in vivo studies conducted in this research demonstrate that BPQDs-DOX@OPM effectively delivers the drug to the tumor site with prolonged circulation time and targeted delivery. Moreover, the combined therapy using BPQDs-DOX@OPM exhibits stronger anti-tumor activity than single-agent chemotherapy. Notably, the BPQDs-DOX@OPM drug delivery system demonstrates high biosafety.

In conclusion, this study presents a non-toxic, long-circulation, targeted delivery chemotherapy-PTT combined drug delivery system for the treatment of OS. The findings highlight the potential of BPQDs-DOX@OPM as a novel therapeutic strategy for enhancing the therapeutic outcomes of OS treatment.

## Supplementary Information

**Additional file 1: Figure S1.** BPQDs, OPM, BPQDs@OPM, BPQDs-DOX@OPM Zeta Potential. **Figure S2.** BPQDs, OPM, BPQDs@OPM, BPQDs-DOX@OPM particle size. **Figure S3.** DOX concentration standard curve. **Figure S4.** Relative cell survival rate of each component of BPQDs@OPM system after co-incubation with Saos-2 cells for 24h.

## Data Availability

The datasets used and analyzed during the current study are available from the corresponding author upon reasonable request.
