# Supplementary material for: Black phosphorus quantum dots camouflaged with platelet-osteosarcoma hybrid membrane and doxorubicin for combined therapy of osteosarcoma
Source: J Nanobiotechnology. 2023 Jul 28;21:243. doi: 10.1186/s12951-023-02016-9 (PMC10386629; doi:10.1186/s12951-023-02016-9)
Supplement: Supplementary file 1 — Additional file 1: Figure S1. BPQDs, OPM, BPQDs@OPM, BPQDs-DOX@OPM Zeta Potential. Figure S2. BPQDs, OPM, BPQDs@OPM, BPQDs-DOX@OPM particle size. Figure S3. DOX concentration standard curve. Figure S4. Relative cell survival rate of each component of BPQDs@OPM system after co-incubation with Saos-2 cells for 24h. [file 12951_2023_2016_MOESM1_ESM.docx]

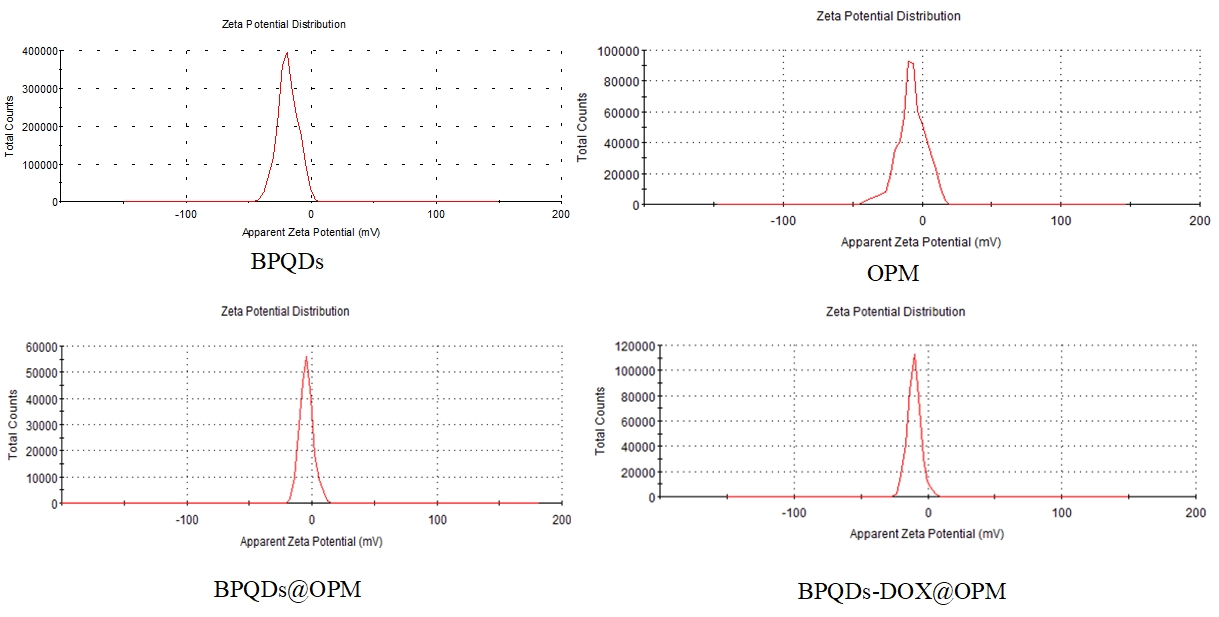


Figure S1, BPQDs, OPM, BPQDs@OPM, BPQDs-DOX@OPM Zeta Potential


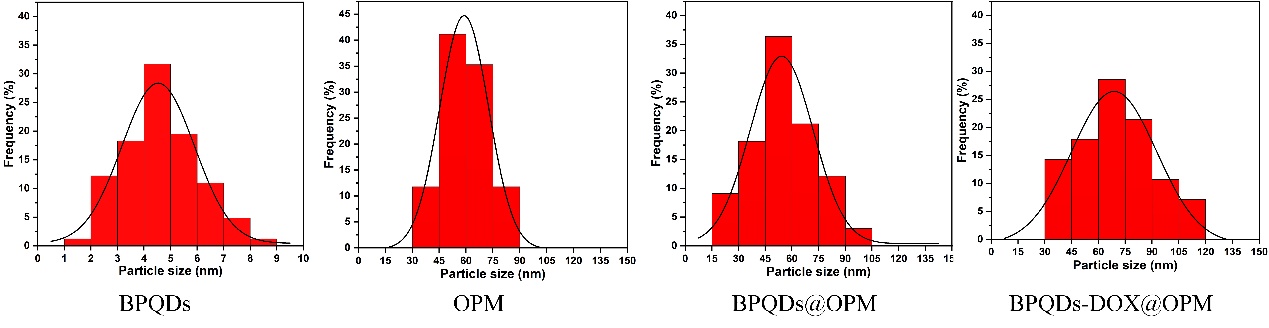
As shown in Figure 1, the zeta potential of BPQDs measured by DLS was -18.07 ± 0.46 mv; the zeta potential of OPM hybrid membrane was -9.43 ± 0.78 mv, the BPQDs@OPM zeta potential was -4.64 ± 0.34 mv, and the zeta potential of BPQDs-DOX@OPM combined drug delivery system was -7.70 ± 0.46 mv. BPQDs-DOX@OPM Zeta potential was close to that of OPM.

Figure S2, BPQDs, OPM, BPQDs@OPM, BPQDs-DOX@OPM particle size

As shown in Figure 2, the particle sizes of each group were measured according to the transmission electron micrographs, and the particle sizes of BPQDs, OPM, BPQDs@OPM, and BPQDs-DOX@OPM were 4.53 ± 1.36 nm, 58.96 ± 13.65 nm, 54.22 ± 17.69 nm, and 68.80 ± 23.78 nm, respectively. the particle sizes of BPQDs-DOX@OPM were close to the OPM hybridized membrane particle size.


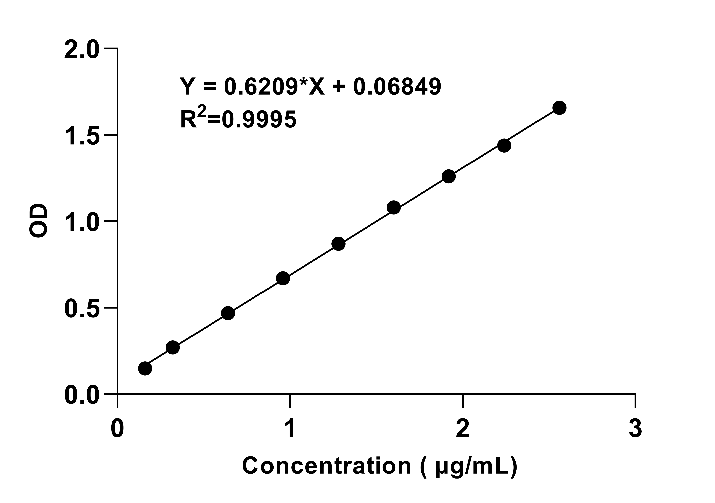
Figure S3. DOX concentration standard curve


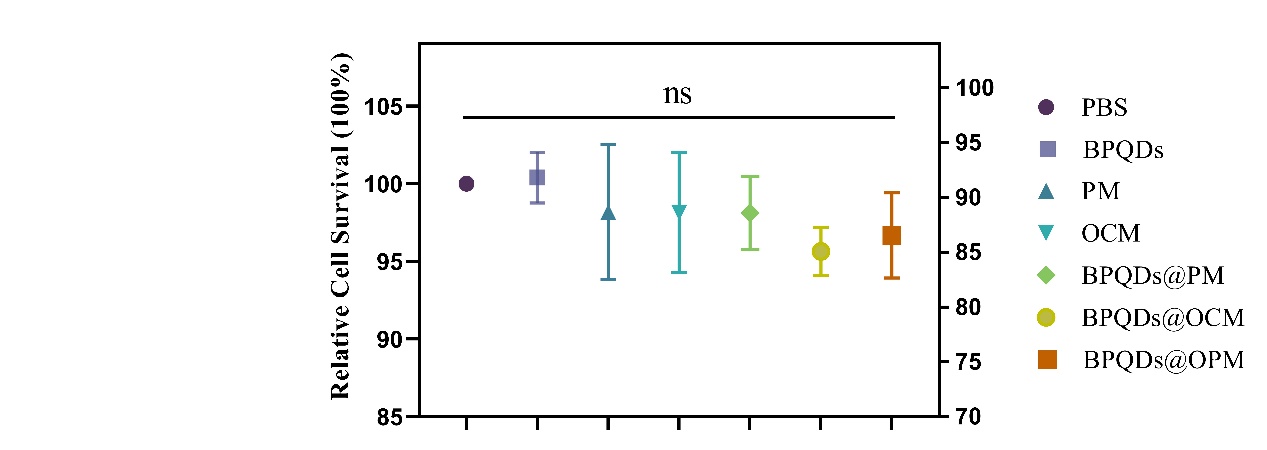
As shown in Figure 3, the linear regression equation of the standard curve of DOX concentration was: Y = 0.6209*X + 0.06849 (R^2^= 0.9995), and the results showed that there was a good linear relationship between OD and DOX concentration in the concentration range of 0.16-2.56 μg/mL.

Figure S4. Relative cell survival rate of each component of BPQDs@OPM system after co-incubation with Saos-2 cells for 24h

As shown in Figure 4, the results of the proliferation activity assay of CCK-8 cells after 12 h of BPQDs@OPM treatment of Saos-2 cells plating showed that the relative survival rate of cells in each group was maintained above 95% after 24 h of co-incubation of each component of the drug delivery system with cells.
